# Eliminating the Drones

**Published:** 1914-06

**Authors:** 


Eliminating the Drones.—In the opinion of the *American Journal of Clincial Medicine,* the medical profession of this country is in a state of lethargy from which it needs awakening. That journal says "the astounding indifference with regard to the future, the absence of fighting spirit, the willingness to submit rather than to stand up in defense of our rights is something hard for the ordinary non-professional citizen to understand," and anticipates that the many members of the profession are "bound to be starved out" unless something be quickly done to prevent it. It is a matter of grave doubt whether the medical profession, or any other profession, can insure living compensation for services rendered in any other way except on a basis of merit and of valuable services rendered.

The professions occupy an ethical position entirely different from that of the labor organizations of the country. Labor unions can get together and make their demands upon employers and upon State legislators. Individuality has largely been lost among the laboring classes, and the organization clearly stands superior to the individual. When an individual member of a labor organization falls behind in the general average he can still be carried along by the organization and receive an equal share of compensation with those who are more capable. In the medical and other professions, capability is the measure of compensation, and uneducated, incapable, non-progressive physicians must inevitably fall behind and lose out as members of the profession.

The sooner these incapables are discovered and are forced by their inability to make a living to drop from the ranks of the medical profession, the better it will be for those who depend upon individual merit for success.

In contending for the ethics of the profession, physicians have been too prone to overlook the fact that there are hundreds of mental drones who are hanging on to the profession and attempting to cover up their own lack of qualifications. It isn't that the profession, as a whole, is lethargic, but that individual members in great numbers are absolutely indifferent to professional progress. Those physicians who realize that important and far-reaching discoveries are always being made in medicine, and who study all the while to keep themselves well abreast would perhaps be astonished to know what a large proportion of the physicians of this country regard themselves really as "finished" in their studies as soon as they obtain a diploma and a license to practice medicine.

What the profession needs more than organization for the purpose of insuring a living for its members is organization to drive the drones from its ranks, and one that has the courage to eliminate from its membership those who are known to be incapable.

The man professionally unfit for his work lowers the standard of the profession, lessens the esteem in which it is held and makes it more difficult for the man of real merit to succeed.

While properly appreciating the advantages of organization, and of co-operation to secure proper legislative protection and passage of such laws as will benefit the entire country, the Texas Medical Journal is inclined to doubt the expediency of seeking such legislation as will increase by law the revenue to be derived from the practice of medicine.

Such efforts appear selfish, and physicians cannot afford to be selfish. They must be broad and liberal, for they are real professional benefactors, and should the public ever come to regard them as serving humanity solely for the money, the esteem in which the profession has always been held will be greatly lessened. Of course, physicians must make money, but money-making should never be their ultima Thule. There is little doubt that the really competent, progressive physician who is always a student will succeed, but he will succeed because of individual effort, rather than by reason of legislative help.

---

Grand Rapids, Michigan, is to have a half million dollar hospital, a gift to the city from Mr. John W. Blodgett. The work on the building is now under way.

